# Hemobilia: Perspective and Role of the Advanced Endoscopist

**DOI:** 10.1155/2018/3670739

**Published:** 2018-07-12

**Authors:** Rani Berry, James Han, Mohit Girotra, James H. Tabibian

**Affiliations:** ^1^Department of Internal Medicine, Ronald Reagan UCLA Medical Center, Los Angeles, CA, USA; ^2^Department of Internal Medicine, University of California, Irvine, CA, USA; ^3^Division of Gastroenterology, University of Miami, Miller School of Medicine, Miami, FL, USA; ^4^Division of Gastroenterology, Department of Medicine, Olive View-UCLA Medical Center, Sylmar, CA, USA

## Abstract

Hemobilia refers to bleeding from and/or into the biliary tract and is an uncommon cause of gastrointestinal hemorrhage. Hemobilia has been documented since the 1600s, but due to its relative rarity, it has only been more critically examined in recent decades. Most cases of hemobilia are iatrogenic and caused by procedures involving the liver, pancreas, bile ducts, and/or the hepatopancreatobiliary vasculature, with trauma and malignancy representing the two other major causes. A classic triad of right upper quadrant pain, jaundice, and overt upper gastrointestinal bleeding has been described, but this is present in only 25–30% of patients with hemobilia. Historically, the gold standard for diagnosis and treatment has been angiography and interventional radiologic intervention, respectively. However, the paradigm is shifting, at least in select cases, towards first-line reliance on noninvasive imaging (e.g., computed tomography) and therapeutic endoscopy, owing to advances in and the less invasive nature of both, while saving interventional radiological and/or surgical intervention for refractory or imminently life-threatening cases.

## 1. Introduction

### 1.1. Overview

Hemobilia, in the most elemental sense, refers to the admixture of blood in bile or to blood in the biliary tract. The most common causes of hemobilia are surgical and nonsurgical trauma, malignancy, and/or cholangiovenous (or arterobiliary) fistulae. Though hemobilia remains an uncommon cause of digestive tract bleeding, its incidence has gradually increased as the arsenal of advanced endoscopic and other minimally invasive hepatopancreatobiliary procedures has expanded. Likewise, with more hepatopancreatobiliary procedures comes the advent of new therapeutic endoscopic and interventional radiologic approaches to diagnose and treat hemobilia [1].

### 1.2. Historic Background

**Figure 1:**
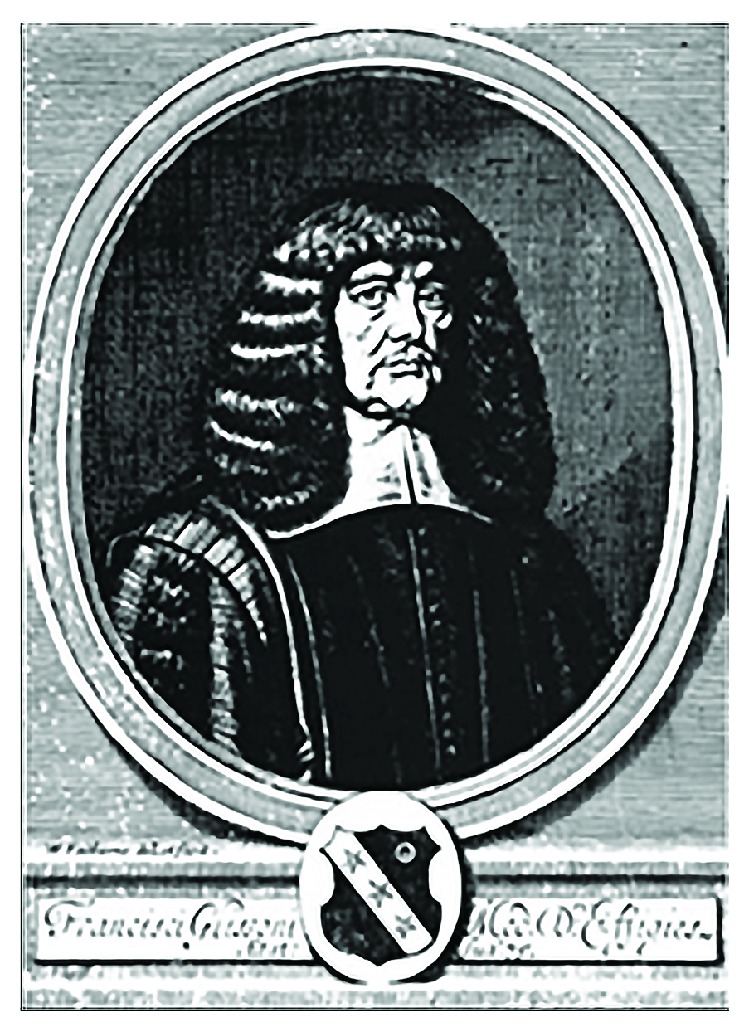
Francis Glisson, who recounted the first recorded case of hemobilia in his treatise *Anatomica Hepatis* [67].

The first known report of hemobilia was from Francis Glisson (Figure 1) in 1654, who described the clinical presentation of a nobleman whom in the midst of a sword fight suffered a fatal blow to the right upper quadrant, leading to massive (upper) gastrointestinal bleeding and death 1 week later. Post mortem, the source of bleeding was found to be from a liver laceration, which in turn led to the landmark description of hemobilia. Antonie Portal was the first to publish a case of hemobilia identified antemortem, reporting suspected hemobilia that was later confirmed on autopsy in 1777. Portal drew important attention to the difficulty in identifying the pinpoint source of bleed, a problem still faced today [2]. One hundred years later, Quincke identified the clinical triad of right upper quadrant pain, jaundice, and upper gastrointestinal tract bleeding, known as Quincke's triad [3]. By the 1900s, there were many scattered case reports of biliary tract hemorrhage, though the term hemobilia was not actually coined until 1948 in a paper entitled, “Hemorrhage into the Biliary Tract Following Trauma: Traumatic Hemobilia” [4].

## 2. Epidemiology

**Figure 2:**
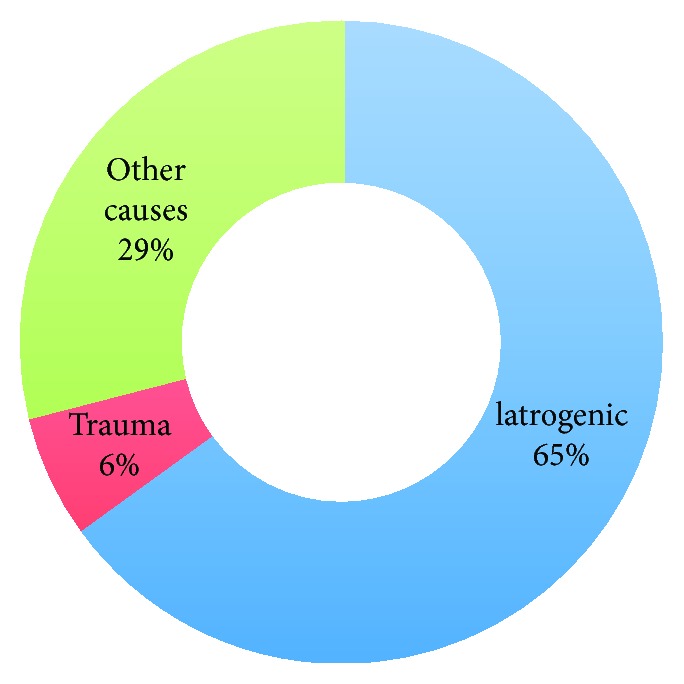
Common causes of hemobilia [6].

Though uncommon, hemobilia is an important cause of upper gastrointestinal hemorrhage. Published data on the topic is mainly in the form of case reports and three large case series. In 1973, Sandblom reported a series reviewing 355 cases and reporting 59 iatrogenic cases (16.6%) and 137 (38.6%) due to trauma [4]. Yoshida et al. published a series of 103 patients in 1987, of whom 41% of cases were iatrogenic and 19% were traumatic, thus reversing the incidence (compared to prior reports) to favor iatrogenic causes, citing increasing hepatobiliary interventional procedures as the primary contributing factor [5]. This sentiment was echoed later on by Green et al. in a series of 222 patients published in 2001, among whom 65% had an iatrogenic cause and only 6% had trauma (Figure 2) [6]. This is consistent with more recent reports indicating iatrogenic injury as the leading cause of hemobilia, accounting for over 50% of all cases.

## 3. Clinical Presentation and Pathophysiology

**Figure 3:**
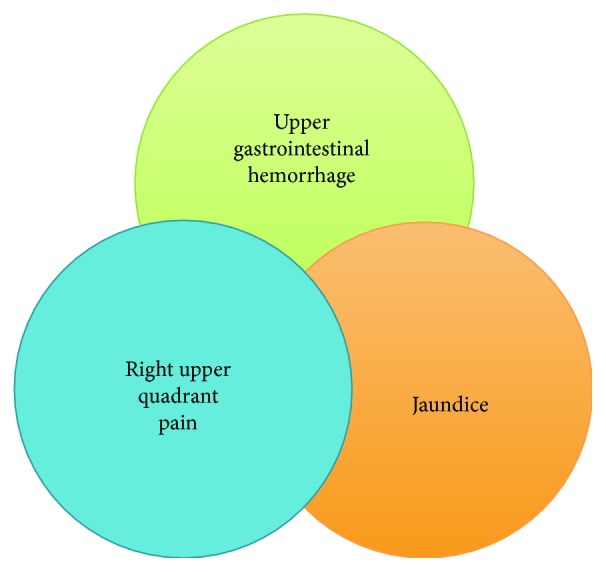
Quincke's triad of hemobilia. Notably, all three symptoms are only present in 22–35% of patients [6, 7].

The classic presentation of hemobilia is formally known as Quincke's triad: jaundice, right upper quadrant abdominal pain, and upper gastrointestinal hemorrhage (Figure 3), but all three findings are only present in 22–35% of cases [6, 7]. The presentation of hemobilia often depends on the cause. For instance, patients with a percutaneous transhepatic biliary drain (PTBD) may present with bloody output from the biliary drain. The timing of presentation can also vary and possibly aid in diagnosis. Endoscopic retrograde cholangiopancreatography- (ERCP-) related hemobilia tends to present immediately or within a few days after the inciting biliary duct injury (e.g., sphincterotomy or biliary stricturoplasty) [8, 9]. In instances where the hepatic artery is ligated during surgery or interventional procedures, an environment for fistulization between a high-pressure arterial system and low-pressure biliary tract (i.e., arteriobilious fistula) is created. The difference in density between blood and bile causes the two to separate once joined within the biliary tree, and as the initial bleed stops, the blood that originally entered the biliary tract begins to clot, serving as a physical impediment to biliary outflow. These clots can cause symptomatic jaundice, biliary obstruction, and subsequent right upper quadrant pain. Clots also cause biliary stasis and hepatobiliary inflammation [8]. As clots travel through the hepatopancreatic ampulla, patients may preferentially complain of discomfort at the level of the right hypochondrium, extending to the epigastrum [8, 10]. Additionally, because of their similar echogenicity, clots can masquerade as biliary stones on imaging studies. Hemobilia can also emanate from venous blood flow, which in contrast tends to be of lower volume or self-limited (except in cases of portal hypertension, in which case it may be of larger volume and/or persistent).

Hemobilia presents on serum laboratory tests as (iron deficiency) anemia and/or as hyperbilirubinemia and elevated alkaline phosphatase and/or aminotransaminases, as seen with other causes of bleeding. Clinically, hemobilia can present as hematemesis, melena, or hematochezia, with or without choluria, depending on the rate of bleeding and anatomical factors (e.g., postbilioenteric surgical anatomy).

## 4. Causes of Hemobilia

The causes of hemobilia have evolved overtime, as alluded to earlier. Possible causes include iatrogenic, traumatic, neoplastic, inflammatory, infectious, and vascular. More recently, iatrogenic causes of hemobilia (though still relatively rare) have superseded other causes in most series/populations and can be categorized by procedural type.

### 4.1. Interventional Procedures

#### 4.1.1. Percutaneous Hepatobiliary Interventions

**Figure 4:**
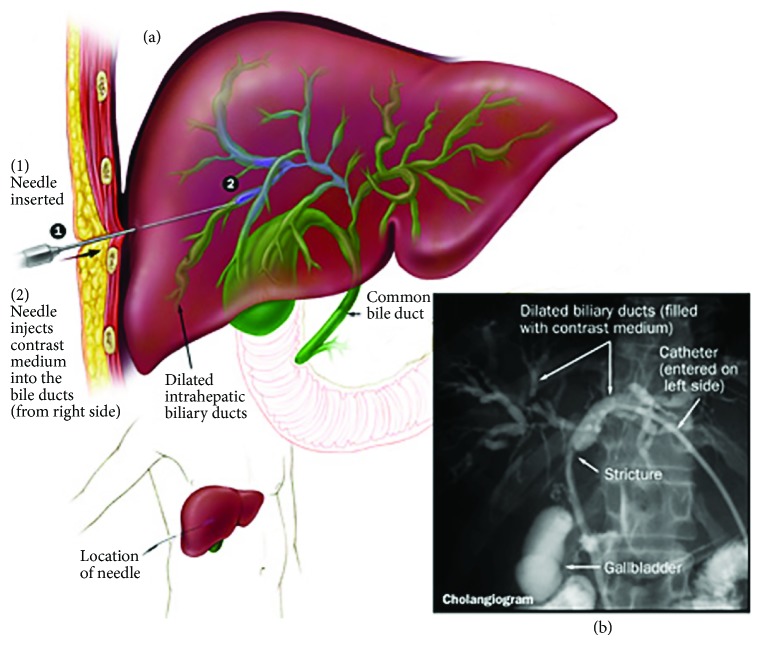
Percutaneous transhepatic cholangiography. This technique can be utilized to inject contrast directly into the biliary system and show, fluoroscopically, evidence of filling defects, which in the proper clinical context may be consistent with (clotted) hemobilia [68].

Common causes of iatrogenic hemobilia include interventions including percutaneous and (to a lesser extent) transjugular liver biopsy, diagnostic percutaneous transhepatic cholangiography (PTC), (Figure 4), and percutaneous transhepatic cholangiography with biliary drain (PTBD) placement; percutaneous biliary therapies such as radiofrequency ablation can also cause hemobilia but collectively comprise a smaller proportion of cases due to their relative rarity [11].

*(1) Percutaneous Liver Biopsy*. The published literature shows some discrepancy in the risk of hemobilia due to liver biopsy; for example, a recent study by Zhou et al. found that “hemobilia accounts for 3% of all major percutaneous liver biopsy complications,” whereas a larger retrospective study reported only 4 cases of hemobilia out of 68,276 liver biopsies (0.005% risk). The discrepancy between studies may suggest that either liver biopsies have become more risky overtime (e.g., due to detection and subsequent sampling of smaller or more central lesions), although another explanation could be that the difference is attributable to better detection and reporting of hemobilia over time, among other potential explanations [12, 13].

*(2) Percutaneous Transhepatic Cholangiography with and without Biliary Drain*. Percutaneous interventions run the risk of inadvertently nicking a vascular structure, resulting in hemobilia, with the risk being higher in the case of factors such as a nondilated biliary tree (i.e., a smaller target). Rivera et al. compared hemobilia caused by PTBD versus PTC and found that the risk of hemobilia is higher with PTBD (2.2%) versus the rate from PTC alone (0.7%) [14]. Thought varies as to the reason for this 3-fold increase in hemobilia with PTBD compared to PTC, but it is likely that at least some of this difference may be due to the greater size of the aperture made in the bile duct wall with PTBD and the presence of a foreign material remaining in the duct which can serve as cause of inflammation or erosion [8]. A retrospective cohort study had similar results, citing that the risk of hepatic artery injury was 2.6% with PTBD and 0.7% with PTC [15]. Less common interventional procedures which may result in hemobilia include ultrasound-guided radiofrequency ablation and transarterial chemoembolization as well as transjugular intrahepatic portosystemic shunt placement [16–20].

#### 4.1.2. Endoscopic Hepatopancreatobiliary Interventions

The main endoscopic procedure associated with hemobilia is ERCP and, in particular, sphincterotomy. Though sphincterotomy-associated bleeding typically occurs at the level of cut papillary sphincter, as initially described by Sandblom, blood can occasionally flow into or reflux from the duodenum back into the biliary tree [4]. In general, the risk of hemobilia depends on the invasiveness of the maneuvers performed during ERCP (e.g., stricturoplasty and extraction of large stones) as well as patient-level variables such as coagulopathy or presence of malignant tissue. Additional risk factors for ERCP-related hemobilia which should be mentioned are variant anatomy, especially anomalous location of the ampulla, aggressive biliary balloon dilation or intraductal biopsying, vascular anomalies (e.g., associated with hereditary hemorrhagic telangiectasia), and transbiliary ductal drainage procedures (e.g., EUS-guided choledochoduodenostomy and hepaticogastrostomy) [8, 21–24].

### 4.2. Noniatrogenic Causes

#### 4.2.1. Portal Biliopathy

Hemobilia can rarely occur due to portal biliopathy, with or without preceding biliary tract intervention. Portal biliopathy occurs as a result of hypertension of the peribiliary (e.g., choledochal) venous plexus, often in patients with portal vein thrombosis and ensuing portal cavernomas, and manifests radiographically or cholangiographically with multifocal biliary stenoses from tortuous venous structures encircling the bile duct. Hemobilia in this context often requires interventional radiologic or other endoscopic or nonendoscopic intervention as the bleeding is not from an abnormality of the biliary epithelium. Risk factors such as coagulopathy and biliary stenosis also increase the risk of ERCP-related hemobilia [25–27].

#### 4.2.2. Chronic Ductal Obstruction

Obstruction of the hepatopancreatobiliary tract can potentially lead to inflammation, erosion, and fistulization with adjacent structures with resultant hemobilia [28]. As mentioned earlier, intrabiliary clots which form due to hemobilia are often mistaken as gallstones; however, even when gallstones are indeed present, there can still be concurrent hemobilia, especially in cases in which the stone erodes through the cystic artery or other vascular structures and cause bleeding, analogous to how a stone can erode and fistulize into the duodenum and cause outlet obstruction in Bouveret's syndrome [29]. It is worth mentioning here, although not classified as hemobilia, that hemosuccus pancreaticus (also referred to as Wirsungorrhagia) can occur via pathophysiologically similar mechanisms, for example, pancreatitis eroding into the splenic artery and causing bleeding into the main pancreatic duct [30, 31].

#### 4.2.3. Malignancy

Arguably the most common natural cause of hemobilia is due to (primary or metastatic) hepatobiliary tumors [32]. The tumor tissue and vasculature both tend to be more friable leading to an increased risk of spontaneous hemorrhage [13]. As mentioned above, treatment of hepatobiliary tumors (e.g., with radiofrequency ablation) can also lead to hemobilia [29].

#### 4.2.4. Infection

The most clinically significant cause of infectious hemobilia is “tropical hemobilia,” a result of parasitic invasion of the biliary tract. Common instigators include the roundworm (*Ascaris lumbricoides*), the Chinese liver fluke (*Clonorchis sinensis*), and the sheep liver fluke (*Fasciola hepatica*). Echinococcal infections can indirectly cause hemobilia in that hydatid cysts may cause inflammation of perivascular tissue, weakening of vessel walls, and/or pseudoaneurysm formation with resultant bleeding into the biliary tract. Divided by region, China, Korea, and Vietnam carry the highest incidence of ascariasis and subsequently higher rates of hemobilia secondary to this infection [29].

### 4.3. Surgical Interventions

Though surgical intervention is now rarely needed in the treatment of hemobilia, it remains an important cause of hemobilia. Complications of both laparoscopic and laparotomic surgeries that are performed near the cystic and right hepatic artery are most prone to lacerating or otherwise manipulating nearby structures in a manner which can lead to hemobilia. Cholecystectomy, liver transplantation, and pancreaticoduodenectomy (Whipple procedure) are examples of surgeries which have been reported to cause hemobilia [29, 33].

## 5. Diagnosis

**Figure 5:**
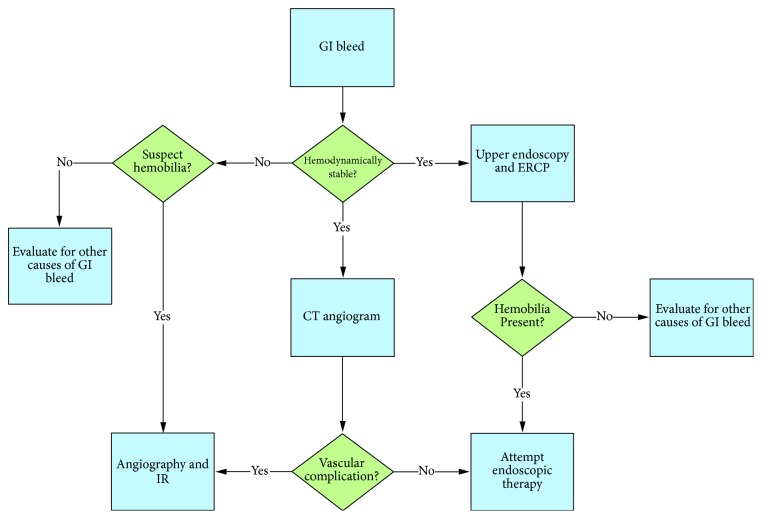
Proposed algorithm for the diagnosis of hemobilia. Vascular complications include hepatic artery aneurysms, pseudoaneurysms, and cholangiovenous or arterioductal fistulae.

Hemobilia should be suspected in any patient with an unclear source of GI bleed, recent blunt force or penetrating trauma to the upper abdomen, or biliary instrumentation or manipulation, particularly in the context of contemporaneous signs or symptoms of biliary obstruction. The diagnosis of hemobilia requires radiographic or endoscopic findings such as direct visualization of blood emerging from the biliary tract or radiographic findings suggestive of intrabiliary hemorrhage (Figure 5).

### 5.1. Computed Tomography

Computed tomography (CT) of the abdomen (preferably angiography protocol) has become a first-choice diagnostic test for hemobilia due to its noninvasive nature, low radiation exposure compared to angiography, rapid results, and diagnostic performance characteristics. CT imaging has improved dramatically over the years such that even subtle salient abnormalities can be identified [30, 34, 35].

### 5.2. Upper Endoscopy and ERCP

**Figure 6:**
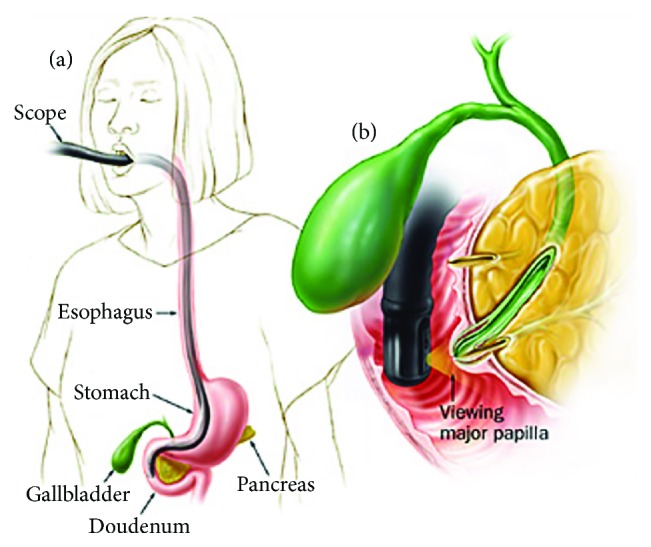
Endoscopic retrograde cholangiopancreatography (ERCP). (a) Schematic of duodenoscope trajectory to reach the major duodenal papilla. (b) Positioning and vantage point of duodenoscope viewing the major duodenal papilla [68].

**Figure 7:**
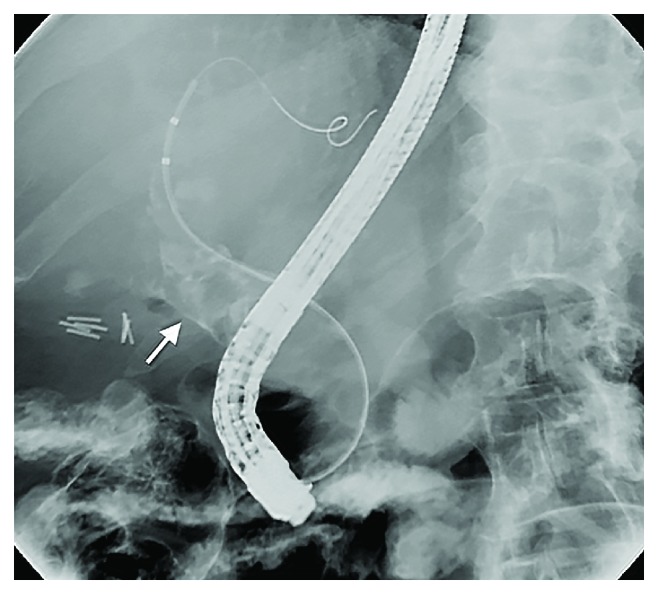
ERCP demonstrating radiolucent filling defects in a dilated CBD in a patient found to have hemobilia. This fluoroscopic image obtained during ERCP is from a patient with recent fine-needle aspiration (FNA) of a malignant-appearing pancreatic head mass one day prior. The radiolucent filling defects throughout the common hepatic duct and more proximal perihilar ducts in the context of recent FNA are consistent with hemobilia.

**Figure 8:**
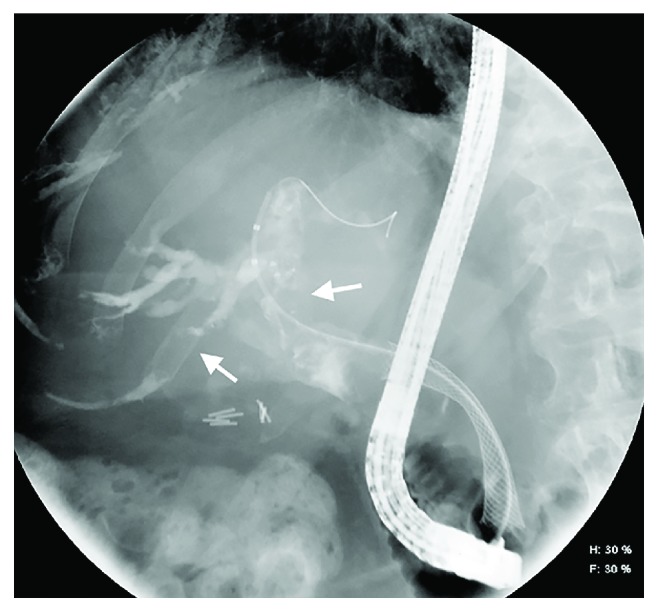
ERCP with therapeutic placement of self-expanding metallic stent (SEMS). A biliary SEMS has been placed during ERCP, which helps mitigate both bleeding and biliary obstruction (from large-volume hemobilia as well as the malignant pancreatic head mass).

Endoscopy is commonly used to evaluate upper gastrointestinal bleeding (with or without suspicion of hemobilia) and can, sometimes incidentally, find hemobilia as the cause/source of bleeding. Up to 60% of hemobilia cases can be diagnosed by endoscopy [35]. Depending on anatomical and other factors, a duodenoscope (i.e., side-viewing scope) may be needed to visualize the ampulla and assess clots or other evidence of bleeding. ERCP can be used to visualize the biliary tree or gallbladder and may offer therapeutic options in patients with hemobilia and/or associated biliary obstruction (Figure 6). Characteristic ERCP findings that suggest the presence of blood clots include amorphous, tubular, or cast-like filling defects; gallbladder filling defects, and otherwise unexplained common bile ductal dilation [36] (Figures 7 and 8).

The presence of gallbladder filling defects does not necessary mean that the gallbladder is the source of bleeding, as blood can enter the gallbladder retrograde from the biliary ducts. If endoscopic ultrasound (EUS) is available, it may be used as an adjunctive noninvasive method to evaluate vascular aneurysms and blood clots within the biliary ducts when ERCP findings are equivocal [36–38]. EUS can also be used to detect portal biliopathy-related bleeding (e.g., in the context of portal hypertension with intra- or paracholedochal varices [30].

### 5.3. Angiography

Although formal angiography is no longer used as first-line study, it remains the gold standard for both diagnosis and treatment of hemobilia in most settings. If the bleeding vessel has not already been identified on noninvasive imaging, the first angiographic study should be a celiac arteriogram with delayed phase imaging to visualize both the hepatic arteries and the portal vein. It is necessary to ensure that the portal vein is patent prior to hepatic artery embolization because the liver is supplied by both the hepatic artery and the portal vein; performing hepatic artery embolization when the portal vein is thrombosed or otherwise obstructed could potentially cause significant hepatic ischemia. This is especially important in patients who are liver transplant recipients as the transplanted liver does not receive as much blood from the portal vein as a native liver, thus making it more dependent on the hepatic artery for its blood supply. Patients with cirrhosis and hereditary hemorrhagic telangiectasia involving the liver are also at risk for hepatic ischemia for the same reason [39].

Any percutaneous drains should be removed over a guidewire (to maintain access) so as to disable the potential tamponade effect from the drain and allow for blood to flow and be visualized angiographically. Evaluation of the vasculature typically progresses in a stepwise fashion; if celiac arteriography does not reveal a clear source, then the catheter should be advanced and arteriographies of both the left and right hepatic arteries should be performed. If no branches of the celiac or hepatic arteries are identified as the source, then the superior mesenteric artery should be interrogated for potential accessory hepatic arteries. Contrast extravasation into the biliary tree, peripheral arterial truncation, arterial transection, pseudoaneurysms, and arterioportal fistula can all also suggest arterial injury [40].

### 5.4. Other Diagnostic Modalities

Lesser used methods include magnetic resonance cholangiopancreatography (MRCP), abdominal ultrasound (US), and surgical exploration. MRCP is a noninvasive alternative to ERCP but lacks the therapeutic options that ERCP offers and also requires more time for image acquisition. Abdominal US has been used to evaluate the presence of blood within the gallbladder, but its diagnostic effectiveness is limited due to its limited ability to visualize the biliary ducts, particular the distal CBD and in patients with truncal obesity. Surgical exploration is usually reserved as a final option in which other modalities are unable to identify or resolve the hemobilia [30].

## 6. Management

Management of hemobilia consists of two main objectives: (1) achieving hemostasis and (2) maintaining bile flow. The latter is important because the formation of blood clots within the biliary tract can cause complications such as obstructive jaundice, acute cholangitis, acute cholecystitis, and pancreatitis [8].

**Figure 9:**
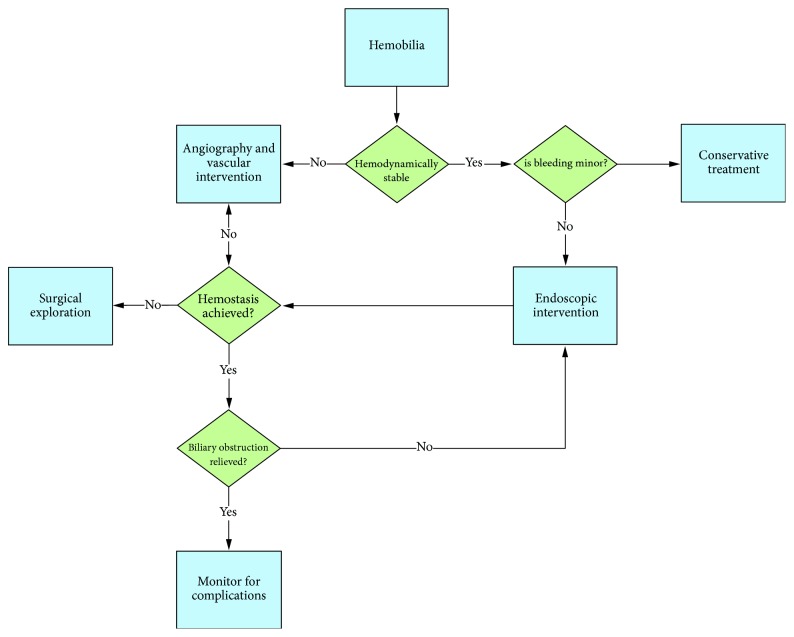
Proposed algorithm for management of hemobilia. Endoscopic intervention includes stenting, endonasal biliary drainage, and various techniques to achieve hemostasis at the ampulla.

The approach to management depends on several factors, including the suspected source of bleeding (arterial versus venous bleeding), degree of hemodynamic instability, and etiology/cause (Figure 9). All patients should have a type and screen performed and be closely monitored for hemodynamic instability. Patients who present with minor hemobilia can potentially be addressed with conservative treatment, including intravenous fluids and correction of coagulopathy. Major hemobilia that causes significant hemoglobin drop or persistent bleeding typically requires endoscopic, radiologic, or, rarely, surgical intervention. Hemodynamically unstable patients should go directly to interventional radiology for hepatic angiogram and embolization or to surgery. Vasopressors may be necessary in cases of major hemobilia as part of resuscitative measures and as a bridge to therapeutic intervention.

### 6.1. Conservative Treatment

Minor hemobilia, which typically presents as blood-tinged output from a biliary drainage catheter, is often due to injury related to PTBD catheters and can often be treated conservatively. Exchanging a PTBD catheter with a larger sized one and adjusting its position such that the side holes of the tube are not in the same location as potential portal vein transgression sites can also help tamponade blood by increasing pressure on the walls of the bile ducts. Any underlying coagulopathies should be corrected as well. Minor hemobilia will often resolve with maturation of the surgically created tract. A tractogram or “tubogram”, an imaging study where contrast is injected into the tract to visualize its course and patency, can be performed if bleeding persists or if there is impaired drainage (e.g., due to obstruction from clot material). If hemobilia persists, options such as embolization of the existing percutaneous tract and creation of a new tract can be considered [39].

### 6.2. Advanced Endoscopic Techniques

For hemodynamically stable hemobilia without clear arterial sources of bleeding or significant vascular abnormalities on noninvasive imaging, upper endoscopy (with a duodenoscope or a clear endcap-outfitted gastroscope) and ERCP are typically the initial therapeutic procedure of choice because of its utility in concurrently managing both bleeding and biliary obstruction [41].

**Figure 10:**
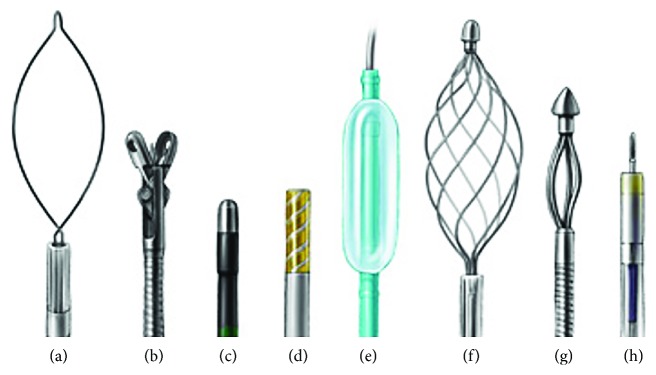
Accessory instruments relevant to hemobilia which may be used through a duodenoscope (or therapeutic gastroscope). (a) Snare, (b) biopsy forceps, (c) heater probe, (d) bipolar probe, (e) dilation balloon, (f) retrieval basket, (g) mechanical lithotriptor, and (h) sclerotherapy needle [68].

**Figure 11:**
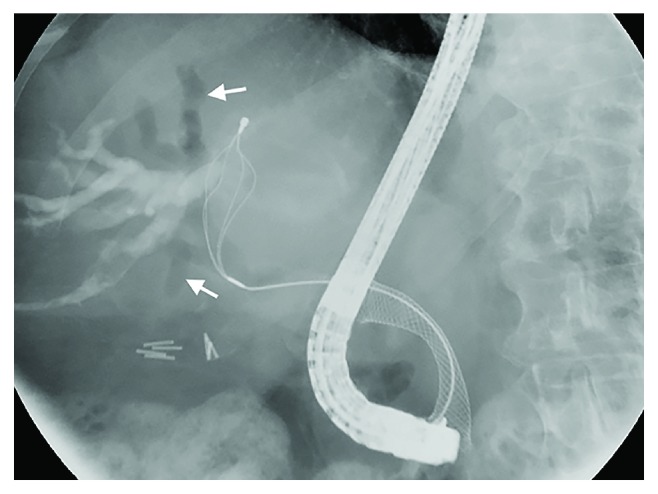
ERCP following several sweeps with flower basket demonstrating clearance of the hemobilia and resultant air cholangiogram. A self-expanding metallic stent is also noted in the biliary tree, which helps maintain luminal patency and flow. Arrows denote intraductal air, indicative of ductal patency (i.e., communication with the duodenum).

There are a wide variety of endoscopic techniques to achieve hemostasis; the choice of which to implement will depend on the cause (e.g., trauma), location (e.g., common hepatic duct), and source (e.g., paracholedochal vein) of hemobilia, for instance, postsphincterotomy hemobilia, which typically is a result of injury to the posterior branch of the superior pancreaticoduodenal artery (itself a branch of the gastroduodenal artery) during sphincterotomy. Management options for hemobilia in this context include spraying diluted epinephrine (1 : 10,000) over the area of hemorrhage, injection of epinephrine into the adjacent tissue, monopolar or bipolar coagulation, fibrin sealant injection, hemoclipping, balloon tamponading, and stent placement (Figure 10) [42–48]. These methods are most useful for postsphincterotomy hemobilia or in cases where the site of bleeding is distal, for example, located at the level of the papilla or ampulla. When hemobilia is from a more proximal (e.g., perihilar) bleeding source, the accessories and methods to treat the hemobilia tend to be different and include devices to extract intraductal clots, for example, extraction balloon catheters and retrieval baskets (Figures 10 and 11), followed by stent placement. One case report has also described the use of endobiliary radiofrequency ablation for hemorrhage secondary to malignant hemobilia [49].

#### 6.2.1. Endoscopic Ultrasound-Guided Biliary Drainage Procedures (EUS-BD)

ERCP-guided drainage using sphincterotomy, biliary stenting, and biliary drain placement is associated with over a 95% success rate and an adverse event rate of 5–10% [50]. The majority of complications arise to difficulties with biliary cannulation. In one study, biliary cannulation was rated as difficult in 15–22% of patients and not achievable in 7–13% of patients, mostly due to cancers of the pancreatic head causing obstruction [51]. Endoscopic drainage has similar rates of success and complications when compared to percutaneous drainage but is often preferred by patients due to its superior comfort [52].

When ERCP-guided drainage cannot be achieved, EUS-guided options for biliary drainage (EUS-BD) can be pursued as an alternative to percutaneous drainage. EUS-BD techniques include EUS-guided choledochoduodenostomy (EUS-CDS) and EUS-guided hepaticogastrostomy (EUS-HGS). When combined with stenting, these procedures are associated with a 90% success rate, and an adverse event rate of 8–25% [23, 24]. The procedure, however, is highly operator dependent and may require specialized training in EUS.

#### 6.2.2. Biliary Stenting

The use of biliary stents merits additional discussion. Stents have been shown to achieve immediate hemostasis in certain cases and work by creating a tamponade effect on the bile wall while maintaining luminal patency and thus bile flow. It can act as salvage therapy when other methods fail and as a bridge to more permanent therapy through interventional radiology or surgery [53]. Both metal and plastic stents has been used successfully for hemobilia resulting from sphincterotomy, ductal dilation for biliary stenosis, fine-needle aspiration (e.g., pancreatic), bile duct biopsy, and malignancy, among other causes. Fully covered self-expanding metallic stents (FCSEMs) appear to have better tamponade and patency and have thus largely supplanted plastic stents when and where available [53–56]. Newer lumen-apposing biflanged FCSEMSs can be used to mitigate the risk of stent migration but at an increased risk of perforation during insertion [57].

Another type, the biodegradable biliary stent, has been proposed for use in hemobilia, but it has only been studied in patients with biliary strictures following hepaticojejunostomy procedures thus far (thereby potentially avoiding the need for cumbersome repeat ERCP); however, the long-term ductal patency after the use of biodegradable stents has not been studied yet [58]. Stenting can also be performed in conjunction with other methods; for example, balloon tamponade can be performed by inserting a biliary dilation balloon catheter into the common bile duct as a temporizing measure until blood flow has slowed adequately enough to permit stent placement as a more durable treatment [45]. In addition, endoscopic nasobiliary drainage can help treat hemobilia, though it is not commonly performed any longer (primarily due to the associated discomfort) and is mainly limited to postliver transplantation patients. An advantage to nasobiliary drainage is that it enables monitoring of bleeding, irrigation of the bile duct, and follow-up cholangiograms without a need for a repeat endoscopy; the same applies to percutaneous biliary drains [36, 59]. When drainage alone is inadequate, infusions of thrombolytic agents directly into the biliary tree via a drain have been described to dissolve biliary blood clots, though this technique requires validation before it can be widely recommended [44].

### 6.3. Transcatheter Arterial Embolization

As the cause of hemobilia has shifted away from traumatic to iatrogenic over the years, radiologic intervention has become the gold standard for both diagnosis and management of persistent or hemodynamically unstable hemobilia. Angiography with transcatheter arterial embolization (TAE) should be considered as the initial therapy of choice if noninvasive imaging shows significant arterial extravasation, the presence of large arterial aneurysms or pseudoaneurysms, presence of arteriobiliary fistulae, and/or intrahepatic or extrahepatic vascular lesions. The success rate of TAE has been reported to be as high as 80% to 100% [60, 61]. TAE should be avoided, however, in patients with liver allografts, cirrhosis with concurrent shock, and portal vein thrombosis given that these patients have compromised collateral blood flow from the portal vein, as a result of which TAE can lead to ischemic liver injury [30]. Such patients may benefit from arterial stenting (as a tamponading measure) instead.

Once the bleeding site has been identified angiographically, superselection of the injured artery via threading of a microcatheter to the target area is performed, followed by embolization using coils. Coiling should be deployed in a distal-to-proximal fashion to ensure that no back bleeding occurs via intrahepatic arterial collaterals [39]. Pseudoaneurysms should be embolized with coils from the two ends to reduce the risk of enlarging the aneurysm. Alternatives to coils include embolization with Gelfoam, PVA particles, and liquid embolic agents such as n-BCA, Onyx, or thrombin. There have also been case reports of percutaneous injection of thrombin into pseudoaneurysms under ultrasound guidance [62]. The method of embolization depends on the anatomy of the hepatic arteries, presence of vasospasms, tortuosity of vessels, and operator/center experience. For instance, liquid embolic agents may be helpful in patients with tortuous vessels or when there are several smaller feed into an aneurysm but require experienced radiologists due to the risk of spilling the agent and causing embolization of nontarget arteries or the biliary ducts [63].

If selective embolization of the bleeding artery cannot be performed, nonselective embolization of the left or right hepatic artery may be performed. In patients who are hemodynamically unstable, embolization of the main hepatic artery can be performed if the patient is a poor surgical candidate, though recognizing the increased risk of liver necrosis [39]. It is currently not recommended to empirically embolize any hepatic arteries if no bleeding source is detected due to this very risk, even with patent portal veins. Furthermore, because the bile ducts are supplied primary by the hepatic arteries rather than the portal vein, there is a risk of biliary ischemia and resultant multifocal strictures [7].

Complications of TAE include hepatic abscesses, postembolization syndrome, hyperaminotransaminasemia, hepatic ischemia, and hepatic infarction or rarely failure [35]. A study of 72 patients who underwent TAE showed that 55 experienced hepatic ischemia evidenced by transiently elevated serum liver enzymes, while 3 experienced focal hepatic infarcts in the areas corresponding to the embolized arterial branches [61].

### 6.4. Vascular Stenting

An alternative to embolization, as alluded to earlier, is the placement of a covered stent across the site of vascular injury. Stenting has the advantage of preserving flow through the artery, which may be beneficial if not crucial in patients with liver transplants or compromised portal vein flow. The diameter of most hepatic vessels is similar to the size of coronary vessels, making coronary stents ideal for this application. Stent diameter should be slightly oversized by about 10–20% of the diameter of the target vessel and extend approximately 10 mm to either side/end of the site of injury to ensure proper tamponade. There are also new flow-directing stents that reduces flow across the stents into pseudoaneurysms while preserving laminar flow [64, 65].

### 6.5. Surgery

Surgical intervention is rarely necessary and usually reserved for failed endoscopic, endovascular, and/or percutaneous therapies. However, it is a first line if pseudoaneurysms are infected or if they are compressing other vascular structures. Surgery may also be indicated if cholecystitis is present, among other uncommon scenarios. Options for surgery include hepatic artery ligation, pseudoaneurysm excision, or hepatic segmentectomy/lobectomy with the potential for concurrent cholecystectomy if cholecystitis is present or the gallbladder neck is involved. Although surgery has a high success rate of up to >90%, it is also associated with a high mortality of up to 10% [7].

### 6.6. Managing Complications

Complications that arise from hemobilia should be managed as they would be in any other scenario. For example, cholecystitis should be treated with early cholecystectomy, as it carries a high mortality rate with rates of gallbladder perforation between 2–15% [66]. Acute pancreatitis is another complication that can occur due to obstruction of the ampulla or more proximal main pancreatic duct by blood clots and reverse flow of blood into the pancreatic ducts and should be managed medically and in some instances by ERCP. Biliary strictures can form following hepatic artery embolization because the vascular supply for the biliary tree comes mostly from the hepatic artery and these will generally require treatment with endoscopic or percutaneous balloon dilation [7].

## 7. Conclusion

Hemobilia is an unusual but important cause of GI bleeding and most commonly due to hepatopancreatobiliary tract procedures, regional trauma, and malignancy. CT angiography and endoscopy/ERCP have become common initial diagnostic testing modalities due to their versatility in excluding other causes of bleeding, low contrast requirement, and relative safety. Most cases of minor hemobilia can be treated conservatively or with minimally invasive endoscopic management. Major hemobilia which is refractory to conservative measures or leading to hemodynamic instability should be managed by interventional radiology in conjunction with endoscopy/ERCP. While TAE is a mainstay, vascular stenting has gained traction as an alternative to arterial embolization due to the preservation of hepatic arterial blood supply. Surgery is typically reserved as a last resort due to its high mortality rate and invasive nature. Although the gold standard for management remains angiography, with new technologies and techniques such as advanced endoscopic procedures, endoscopy has become an attractive alternative for both the diagnosis and treatment of hemobilia.
